# Dynamics of phytoplankton community in scallop farming waters of the Bohai Sea and North Yellow Sea in China

**DOI:** 10.1186/s12862-022-02002-z

**Published:** 2022-04-15

**Authors:** Ning Kong, Zhaoqun Liu, Zichao Yu, Qiang Fu, Huan Li, Yukun Zhang, Xiao Fang, Fuchong Zhang, Chao Liu, Lingling Wang, Linsheng Song

**Affiliations:** 1 Dalian Key Laboratory of Aquatic Animal Disease Prevention and Control, Dalian Ocean University, Dalian, 116023 China; 2 Functional Laboratory of Marine Fisheries Science and Food Production Processes, Qingdao National Laboratory for Marine Science and Technology, Qingdao, 266235 China; 3 Ocean Fisheries Science Research Institute of Hebei Province, Qinhuangdao, 066201 China; 4 Liaoning Key Laboratory of Marine Animal Immunology and Disease Control, Dalian Ocean University, Dalian, 116023 China; 5 Liaoning Key Laboratory of Marine Animal Immunology, Dalian Ocean University, Dalian, 116023 China

**Keywords:** Phytoplankton community, 18S rDNA, Nutrient limitation, Scallop

## Abstract

**Background:**

As the major suppliers of food for higher consumers, phytoplankton are closely related to the yield, nutritional ingredients and even toxin contents of mariculture animals, potentially influencing the human health when they are consumed. With the increase of shellfish culture density, phytoplankton in the coastal waters have been excessively consumed in recent years, and the nutrients they depend on are becoming more and more limited, which severely restrict the shellfish mariculture and threaten the coastal ecosystems.

**Results:**

In the present study, nutrient concentrations, phytoplankton assemblages and scallop growth status were investigated in the main bay scallop farming waters of the Bohai Sea (Qinhuangdao site) and North Yellow Sea (Zhuanghe site) in 2018. Both phosphate and silicate limitations were observed at the two sites, with the major determinant of phytoplankton assemblages being silicate in Qinhuangdao and phosphate in Zhuanghe, respectively. The phytoplankton assemblages at the two sites displayed different community structures and succession patterns. The phytoplankton community was dominated by dinoflagellates and diatoms in Qinhuangdao, while dinoflagellates were the most abundant group in Zhuanghe, which accounted for 41.9% of the total phytoplankton abundance. The dominant genera of diatoms in Qinhuangdao were *Skeletonema*, *Thalassiosira* and *Leptocylindrus*, while those in Zhuanghe were *Thalassiosira* and *Cyclotella*. Greater biomass and more appropriate structure of phytoplankton contributed to higher growth rate and glycogen content of cultured bay scallops.

**Conclusions:**

Our study characterized the relationship between nutrient concentration, phytoplankton community and scallop mariculture in the main bay scallop farming waters in northern China. The results suggest that, as nutrient limitation intensified, dinoflagellates are becoming the dominant phytoplankton species in the scallop farming waters of the Bohai Sea and the North Yellow Sea, which is harmful to the coastal mariculture.

**Supplementary Information:**

The online version contains supplementary material available at 10.1186/s12862-022-02002-z.

## Background

Phytoplankton, the primary producers in the marine ecosystem, play an indispensable role in energy conversion, nutrient cycling and food web dynamics [5, 8]. As major suppliers of food for higher consumers, the biomass of phytoplankton is closely related to the survival, growth and hence overall yield of mariculture animals, such as shellfish, shrimp and fish [8, 15, 45]. In addition, the community structure of phytoplankton has a great impact on the tastes, nutritional ingredients and even toxin contents of mariculture animals via feeding processes, which ultimately affects the health of humans when they are consumed [15, 19, 26]. Therefore, it is of great economic and ecological significances to explore the dynamics of phytoplankton assemblages in the farming waters.

It has been reported that the biomass and community structure of phytoplankton can be impacted by physical, chemical and biological factors in the farming waters such as water temperature, nutrients and bivalve grazing [34]. Of note, nutrients including nitrogen, phosphorus and silicon are necessary for the basic composition of phytoplankton, controlling the growth and proliferation of phytoplankton in aquatic systems [18]. Nitrogen and phosphorus are required for biosynthesis of many molecules such as amino acids, nucleic acids and lipids, while silicon is involved in building the outer cell wall or frustule of diatoms [28]. The depletion of these nutrients, so-called nutrient limitation, will not only restrict the growth rate of phytoplankton cells, but also drive the succession of phytoplankton assemblages [22, 24, 36, 40]. For example, picophytoplankton *Synechococcus* has outcompeted diatoms and dinoflagellates to be the dominant species in nutrient-limited shelf waters of the Northeast Atlantic [35]. A shift in phytoplankton dominance from diatoms to dinoflagellates was observed in shellfish farming waters of the Zhangzi Island, which was attributed to the phosphate and  silicate limitations [22]. Moreover, the filter feeding of bivalves such as scallops, oysters and mussels, also has a considerable top-down control on the phytoplankton biomass [10, 22]. Their selective feeding on certain algae species would further enhance the phytoplankton succession, making the phytoplankton dynamics more complex in the farming waters [9, 22].

**Fig. 1:**
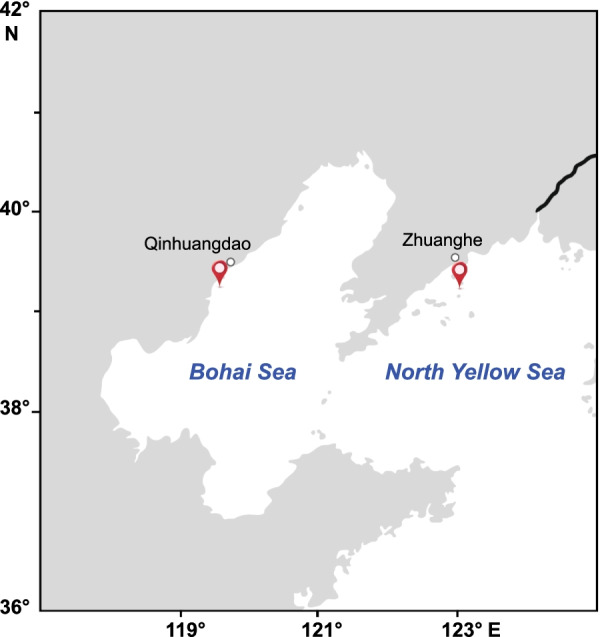
Location of the two sampling sites (red mark, sourced from a map of China, version GS (2019) 1694)

As the main food source for mariculture animals, the succession of phytoplankton determines their yield and quality to a large extent. It has been reported that the large blooms of dinoflagellates or cyanobacteria could induce shrimp mortality or growth diminution [1]. The shift in phytoplankton community from diatoms to dinoflagellates was closely related to the frequent occurrence of Yesso scallop's high mortality in summer [47]. Bay scallop *Argopecten irradians irradians* is one of the dominant cultured shellfish species in northern China [14, 48]. In recent years, the cultured bay scallops exhibit a typical characteristic of "low fatness and high mortality", which is speculated to be related to the biomass shortage and community imbalance of phytoplankton caused by the increasing farming density [17]. In the present study, a comparative analysis of phytoplankton assemblages was conducted in two representative scallop farms located in Qinhuangdao (site Q, Bohai Sea) and Zhuanghe (site Z, North Yellow Sea) in northern China (Fig. 1), with the main objectives to (1) cognize the composition and succession of phytoplankton community in bay scallop farming waters, (2) explore the relationship between phytoplankton community and environmental factors, especially the nutrient status in the water column, and (3) assess the potential effects of phytoplankton community on the yield and quality of bay scallops.

## Results

### Environmental parameters

**Fig. 2:**
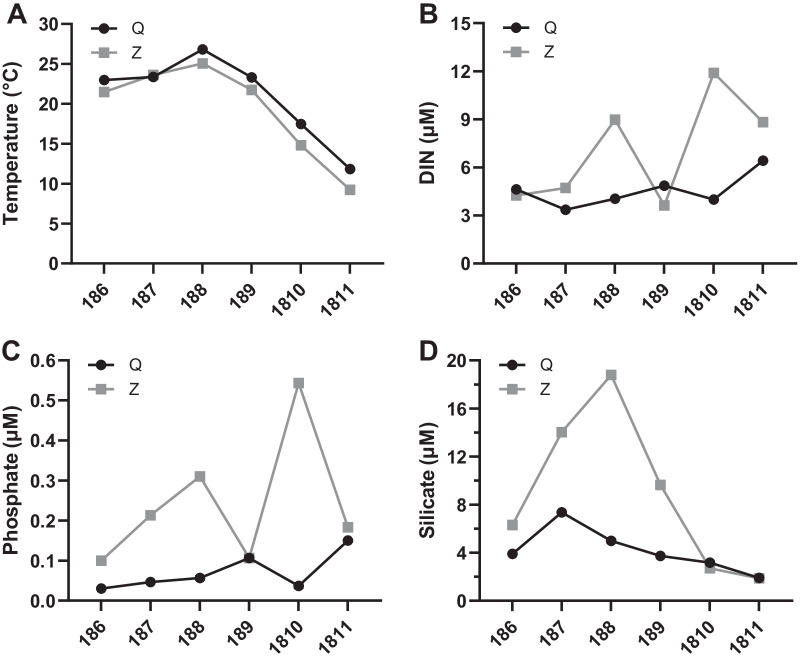
Temporal variation of the environmental parameters. Water temperature (**A**) and concentrations of DIN (**B**), phosphate (**C**) and silicate (**D**) at sites Q and Z

Water temperature, salinity and pH at the two farming sites were recorded from June to November 2018. Similar temporal pattern of water temperature was detected at the two sites, ranging from 11.8 to 26.8 °C at site Q and from 9.2 to 25.1 °C at site Z, respectively (Fig. 2A). No drastic fluctuations were detected in salinity or pH at the two sites (site Q: salinity from 29.8 to 32.4, pH from 8.0 to 8.2; site Z: salinity from 29.4 to 31.2, pH from 8.1 to 8.3; Additional file 1: Figure S1).

The nutrient concentrations in the water column showed different temporal variations at the two sites. Overall, the concentrations of dissolved inorganic nitrogen (DIN), phosphate and silicate at site Q were lower and exhibited less fluctuation than those at site Z (Fig. 2B–D). The DIN concentrations at the two sites were much higher than the minimum threshold (1.0 μM) for phytoplankton growth, which ranged from 3.4 to 6.4 μM at site Q and from 3.6 to 11.9 μM at site Z (Fig. 2B). The phosphate concentrations at site Q were far below the minimum threshold of 0.1 μM during most of the sampling period, while those at site Z varied between 0.1 and 0.5 μM, which were higher than the minimum threshold (Fig. 2C). The silicate concentrations at the two sites both increased first and then decreased to the minima. Silicate limitation in terms of absolute concentration (< 2.0 μM) were only observed in November at the two sites, with the values both being 1.9 μM (Fig. 2D).

**Fig. 3:**
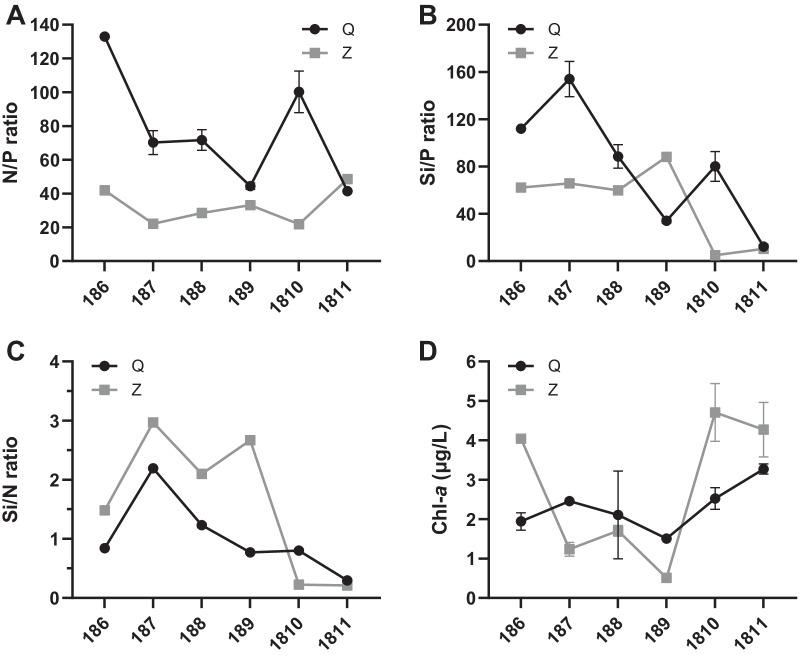
Temporal variation of the nutrient stoichiometric ratios. N/P ratio (**A**), Si/P ratio (**B**), Si/N ratio (**C**) and Chl-*a* concentration (**D**) at sites Q and Z

As for the stoichiometric ratio, the molar ratios of dissolved inorganic nitrogen to phosphate (N/P ratios) at the two sites both diverged significantly from the canonical Redfield N/P ratio of 16. N/P ratios at site Q (ranging from 41.5 to 132.9) were higher than those at site Z (ranging from 21.9 to 48.6) during the sampling period except for November. The highest N/P ratio was observed in June at site Q and November at site Z (Fig. 3A). Similarly, the molar ratios of silicate to phosphate (Si/P ratios) at site Q (ranging from 12.3 to 154.0) were higher than those at site Z (ranging from 4.9 to 88.2) in the sampling months except for September. The Si/P ratios at the two sites showed a downward trend from summer to autumn, with the minimum ratio detected in November at site Q and October at site Z, respectively (Fig. 3B). Likewise, the molar ratios of silicate to dissolved inorganic nitrogen (Si/N ratios) presented a downward trend from summer to autumn at the two sites, where the ratio was higher in July and lower in November (Fig. 3C).

The chlorophyll *a* (Chl-*a*) concentrations at both sites exhibited a downward trend from June to September, followed by a steep increase in October and November. The concentrations of Chl-*a* from July to September at site Q (ranging from 1.5 to 2.5 μg/L) were higher than those at site Z (ranging from 0.5 to 1.7 μg/L), but the situation reversed in the other months (Fig. 3D).

### Phytoplankton composition and succession

High-throughput sequencing of 18S rDNA was applied to estimate the phytoplankton diversity at the two sites. A total of 1334,133 effective sequences were obtained, which were clustered into 3588 sub-operational taxonomic units (sOTUs).

**Fig. 4:**
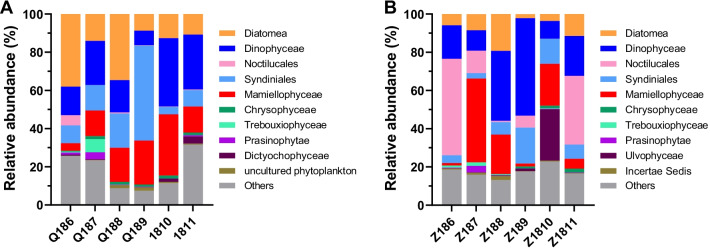
Succession of the phytoplankton communities at sites Q (**A**) and Z (**B**). Phytoplankton composition was represented at level 5 of the taxonomic hierarchy in SILVA v132 release. The top ten abundant groups were shown in the figure and the rest was indicated as “Others”

Taxonomic classification at level 3 showed that Alveolata, Stramenopiles and Chloroplastida were the dominant supergroups in the scallop farming waters, which accounted for 44.4%, 27.1% and 19.7% of the total phytoplankton abundance at site Q, and 59.5%, 12.4% and 22.1% at site Z, respectively (Table 1). At taxonomic level 5, the phytoplankton showed divergent community compositions and succession patterns at the two sites (Fig. 4). At site Q, the phytoplankton community was dominated by Dinophyceae, Diatomea, Mamiellophyceae, and Syndiniales with their average relative abundances of 21.2%, 19.8%, 17.3% and 17.1%, respectively (Fig. 4A). Dinophyceae exhibited a lower relative abundance from June to September, while it became the most dominant taxa in October and November with percentages of 35.8% and 28.7%, respectively. The relative abundance of Diatomea between June and August (average of 28.9%) was higher than that between September and November (average of 10.7%). At site Z, the phytoplankton assemblages were mainly comprised of Dinophyceae, Noctilucales and Mamiellophyceae, which contributed 24.3%, 17.5% and 15.7% of the phytoplankton abundance, respectively (Fig. 4B). The abundance of Dinophyceae was highest in September (51.0%) and lowest in October (9.3%). Noctilucales was the most dominant taxa in June (50.5%) and November (36.0%), whereas its relative abundance was lower in the other months with an average percentage of 4.7%. As for Diatomea, its relative abundance was highest in August (19.3%) and lowest in September (2.2%), with an average of 8.5% during the sampling period.

**Table 1:** Relative abundance of the phytoplankton communities at sites Q and Z

	Q (%)	Z (%)
Alveolata	44.4	59.5
Stramenopiles	27.1	12.4
Chloroplastida	19.7	22.1
Cryptomonadales	4.7	3.1
Prymnesiophyceae	1.7	0.9
Picomonadida	0.9	1.0
Kathablepharidae	0.2	0.3
Others	1.3	0.7

Phytoplankton composition was represented at level 3 of the taxonomic hierarchy in SILVA v132 release. The top seven abundant groups were shown in the table and the rest was indicated as “Others”

**Fig. 5:**
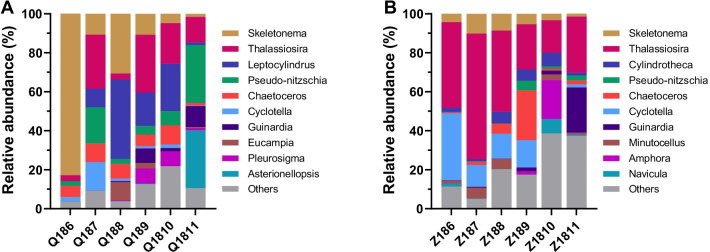
Succession of Diatomea communities at site Q (**A**) and Z (**B**). Phytoplankton composition was represented at level 8 of the taxonomic hierarchy in SILVA v132 release. The top ten abundant groups were shown in the figure and the rest was indicated as “Others”

Diatomea, the preferred food source for bay scallops, was further classified at a higher taxonomic level (Fig. 5). At site Q, the dominant populations of Diatomea were sorted as *Skeletonema* spp., *Thalassiosira* spp. and *Leptocylindrus* spp., which accounted for 23.6%, 16.2% and 15.5% of the total abundance, respectively (Fig. 5A). At site Z, the relative abundance of *Thalassiosira* spp. was absolutely dominant with an average of 36.4% during the sampling period, and *Cyclotella* spp. was the second most abundant population with an average of 12.3% (Fig. 5B).

### Phytoplankton diversity indices

**Fig. 6:**
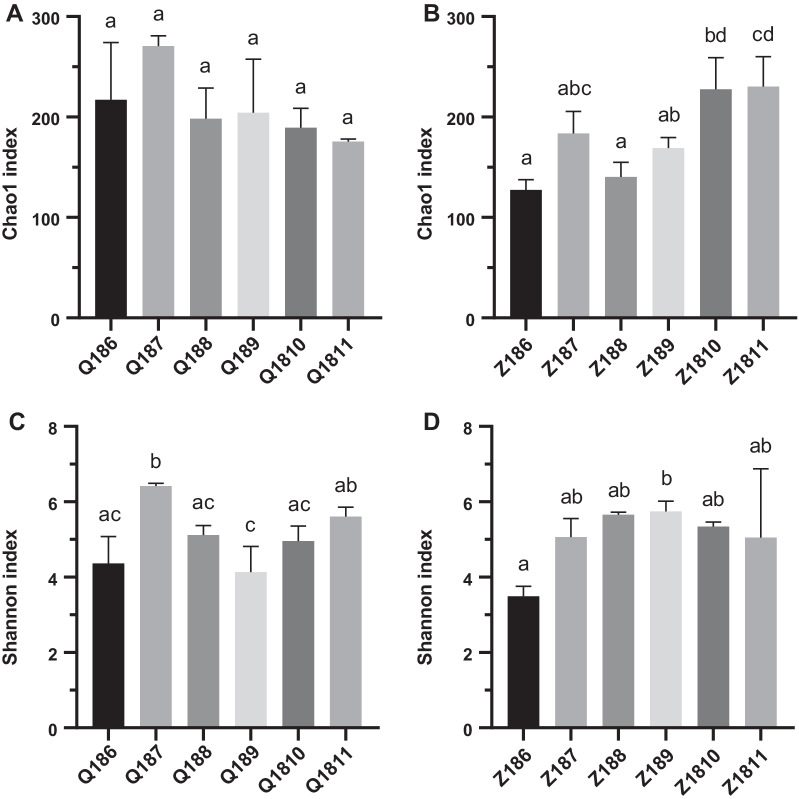
α-diversity indices of phytoplankton communities at sites Q and Z. **A** Temporal variation of Chao1 index at site Q. **B** Temporal variation of Chao1 index at site Z. **C** Temporal variation of Shannon index at site Q. **D** Temporal variation of Shannon index at site Z

The complexity of phytoplankton communities in scallop farming waters was evaluated based on the α-diversity analysis (Fig. 6). The Chao1 index did not change significantly with seasons at site Q, while it changed obviously at site Z (Fig. 6A, B). The Chao1 indices at site Q were higher than those at site Z from June to September, but the situation reversed in October and November. The Shannon index at the two sites fluctuated differently with seasons, which reached the peak in July (6.4) at site Q and September (5.7) at site Z, respectively (Fig. 6C, D).

**Fig. 7:**
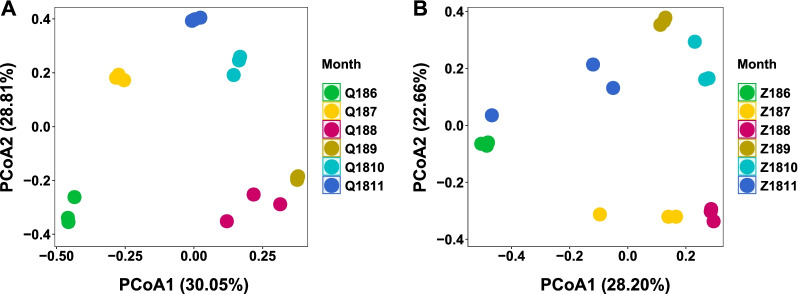
PCoA analysis of the phytoplankton communities at sites Q (**A**) and Z (**B**)

Principal coordinate analysis (PCoA) based on the Bray–Curtis distance was conducted to compare the dissimilarity in phytoplankton community compositions. The first two axes of PCoA explained 58.9% and 50.9% of variation detected at sites Q and Z, respectively. Samples collected in the same month gathered together and separated from other ones (Fig. 7).

### Relationships between phytoplankton communities and environmental factors

**Fig. 8:**
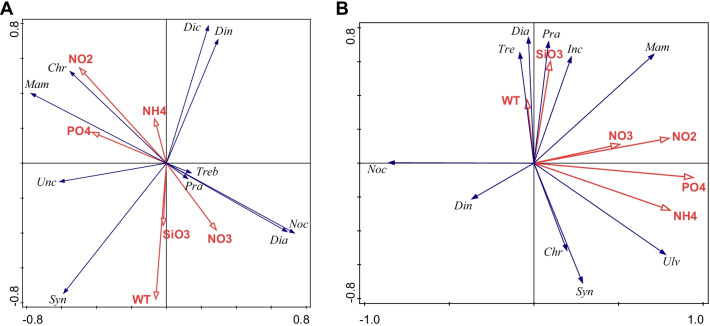
RDA analysis of phytoplankton communities and environmental factors at site Q (**A**) and Z (**B**). Blue vectors: phytoplankton communities, red vectors: environmental factors. *Dia*: Diatomea, *Din*: Dinophyceae, *Noc*: Noctilucales, *Syn*: Syndiniales, *Mam*: Mamiellophyceae, *Chr*: Chrysophyceae, *Tre*: Trebouxiophyceae, *Pra*: Prasinophytae, *Dic*: Dictyochophyceae, *Unc*: uncultured phytoplankton, *Ulv*: Ulvophyceae, *Inc*: Incertae Sedis, WT: water temperature, NH_4_: ammonium, NO_3_: nitrate, NO_2_: nitrite, PO_4_: phosphate, SiO_3_: silicate

Redundancy analysis (RDA) was used to explore the relationships between dominant phytoplankton communities and environmental factors. At site Q, the phytoplankton community was mainly correlated with silicate, water temperature and inorganic nitrogen (Fig. 8A). In particular, the abundance of Diatomea was positively correlated with silicate, water temperature and nitrate, while that of Dinophyceae was negatively correlated with these environmental factors. At site Z, the phosphate, ammonium and nitrite were the main factors that affected the phytoplankton structure (Fig. 8B). Nutrients including phosphate, ammonium, nitrite and nitrate displayed positive effects on the abundances of Mamiellophyceae and Ulvophyceae, whereas they exerted negative effects on those of Dinophyceae and Noctilucales. Moreover, the Diatomea abundance was positively correlated with silicate and water temperature.

### Wet weight and glycogen content of scallops

**Fig. 9:**
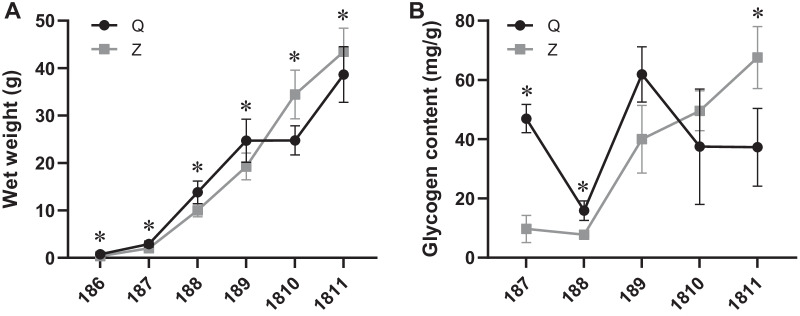
Wet weight (**A**) and glycogen content (**B**) of bay scallops at sites Q and Z. *Significant difference (*p* < 0.05, two-sample *t*-test) between sites Q and Z

During the suspension-culturing period, the wet weight of scallops showed an increasing trend with the sampling months at the two sites. From July to September, the wet weights of scallops at site Q (ranging from 2.9 to 24.7 g) were higher than those at site Z (ranging from 2.0 to 19.3 g), while it was the opposite in October and November (Fig. 9A).

The glycogen content in scallops at the two sites changed significantly with seasons (Fig. 9B). The minimum values of glycogen content were both detected in August (15.9 mg/g at site Q, 7.7 mg/g at site Z) at the two sites, while the maximum values were detected in September (61.9 mg/g) at site Q and November (67.6 mg/g) at site Z.

## Discussion

Phytoplankton are responsible for the vast majority of primary production in marine waters and frequently limit the farming capacity and growth status of filter-feeding bivalves [3]. Numerous studies in different areas have shown that temperature, light and nutrients are important factors impacting the succession of phytoplankton community [25]. In the present study, two scallop farming areas located at almost the same latitude were selected. The temperature and light conditions of the two sites were similar, whereas their nutrient structures were different due to divergent ocean currents and terrestrial inputs between the Bohai Sea and North Yellow sea [43]. A comparative analysis was carried out to explore the difference in phytoplankton assemblages under different nutrient status and its effects on the scallop mariculture.

### The spatiotemporal differences in nutrient status of scallop farming waters

Nutrient limitation exerts a fundamental control on the phytoplankton dynamics and marine food webs. Surface limitation of nitrogen and phosphorus has been observed in many regions, especially in the low-latitude oceans [25]. Absolute concentrations and stoichiometric ratios indicated that there was no nitrogen limitation in the studied scallop farming waters during the sampling period. High levels of nitrogen concentration may be related to the terrestrial input and regeneration of scallop farming by direct excretion of ammonium [31]. The stoichiometric limitation of phosphate was observed in a high frequency, especially in the farming waters of the Bohai Sea with N/P ratio ranging from 41.5 to 132.9. Similar result was obtained by Xu et al. [46] indicating that the Bohai Sea was characterized by phosphate limiting since the 1990s with the average N/P ratio of 22. The higher N/P ratio in the present study indicated that phosphate limitation in the Bohai Sea has become more and more serious, which is not conducive to phytoplankton growth and shellfish mariculture. Compared with phosphate, silicate limitation was observed less frequently in our study, which was only occurred in the autumn at the two sites. The decrease in silicate concentration and Si/N ratio from summer to autumn may be related to scallop farming activities, which could deplete silicate from water column by feeding on diatoms and do not excrete silicate through metabolism [31].

### The response of phytoplankton assemblages to nutrient status

The upper bounds of phytoplankton biomass are determined by the total available amount of nutrients, especially the limited elements [4]. The concentration of Chl-*a*, an indicator of phytoplankton biomass [12], showed a higher mean value at site Z (2.7 μg/L) than site Q (2.3 μg/L), which might be attributed to the higher nutrient concentrations at site Z than site Q. Moreover, a sharp increase in Chl-*a* concentration was observed in October at site Z as the phosphate concentration increased dramatically, proving the decisive effect of limited nutrients on the phytoplankton growth.

It has been reported that phytoplankton succession is largely determined by both concentrations and stoichiometric ratios of nutrients in the water column [3, 38, 46]. Silicate plays a vital role in the dynamics of diatoms, and its effect on the phytoplankton assemblages comes from its influence on the diatom reproduction [7, 49]. At site Q, silicate was the most important factor driving the phytoplankton succession. A shift in phytoplankton dominance from diatoms to dinoflagellates was detected from summer to autumn, which was presumably related to the reduction of Si/P and Si/N ratios during this period. It has been reported that high Si/N ratio favored the growth of diatoms, but they would be replaced by other phytoplanktonic groups that had no requirement for silicate when the Si/N ratio dropped [13, 22, 41]. Changes in nitrogen and phosphorus concentrations have also been considered as important factors promoting the phytoplankton community succession [46]. At site Z, the phytoplankton community was more influenced by phosphate and inorganic nitrogen than by silicate. Of note, dinoflagellates were more prevalent in June, September and November when the N/P ratio was higher than other months. Our results were consistent with the previous report that the increasing N/P ratio was regarded as an important determinant for the replacement of diatoms by dinoflagellates in the Bohai Sea during the last decades [44], since dinoflagellates are less sensitive to low phosphate concentrations than diatoms [6, 22].

### The relationship between phytoplankton assemblages and scallop mariculture

As the important food and energy sources for bivalves, phytoplankton are closely related to the survival and growth of cultured scallops [47]. In the present study, the bay scallops grew faster under higher Chl-*a* concentration, further proving the importance of phytoplankton biomass in the scallop mariculture.

The structure and succession pattern of phytoplankton assemblages, especially the abundance changes of diatoms and dinoflagellates, are considered to be important factors affecting the yield and quality of cultured scallops [47]. Diatoms are high-quality food sources for scallops because they are rich in amino acids, EPA, DHA and sterols, while the dinoflagellates are not favored by scallops because most of them have horny spines and are hard to be digested [29, 37]. Moreover, dinoflagellates (such as Kareniaceae, Peridiniales and Gonyaulacales at sites Q and Z, Additional file 2: Figure S2) can secrete a variety of shellfish toxins mainly including paralytic shellfish toxins and diarrhetic shellfish toxins, which have negative impacts on the growth and health of scallops [20, 27]. In the present study, the phytoplankton community at site Q was characterized by Diatomea-dominated in summer (average of 28.9% from June to August) and Dinophyceae-dominated in autumn (average of 24.0% from September to November). The shift of dominant species from diatoms to dinoflagellates resulted in the lag of scallop growth and also a sharp drop of glycogen content between September and October. Similar result was also reported by Wall et al. [42] that the growth of bay scallop was negatively correlated with densities of dinoflagellates which were more abundant at the most eutrophic site. At site Z, the dominant phytoplankton were dinoflagellates, including Dinophyceae and Noctilucales. Noctilucales, the common red-tide-forming dinoflagellates in coastal waters [33], is harmful to the shellfish mariculture. It not only competes with shellfish for food (especially diatoms), but also adheres to the shellfish gills and affects their filter-feeding and respiration [39]. From June to September, the higher proportion of dinoflagellates and lower proportion of diatoms at site Z than site Q were unfavorable for scallop production, which was reflected by the lower growth rates and glycogen contents of farmed scallops.

Of note, the cultured scallops also bring about changes in the phytoplankton assemblages by determining the scale of grazing pressure [3]. In the intensive farming areas, scallop grazing would lead to a lower phytoplankton biomass and decreased diatom proportion due to their selective feeding [3, 32]. The lower proportion of diatoms than dinoflagellates at sites Q and Z might be resulted from the excessive scallop farming density, which should be strictly controlled in the future scallop mariculture.

## Conclusion

Both phosphate and silicate limitations were observed in the scallop farming waters of the Bohai Sea and North Yellow Sea. The biomass and structure of phytoplankton assemblages were found to vary greatly in different seasons and different sites, owing to the variations in nutrient status, water temperature and scallop grazing scales. Higher growth rate and glycogen content of scallops were supported by greater phytoplankton biomass and appropriate community composition. It is worth noting that dinoflagellates are becoming the dominant phytoplankton species in the scallop farming waters of the Bohai Sea and North Yellow Sea, which indicates the degradation of food web in the coastal mariculture ecosystem.

## Methods

### Sampling sites and sample collection

The present study was carried out from June to November 2018 in two bay scallop farms that located in Qinhuangdao (Site Q, 39° 42′ N, 119° 24′ E) and Zhuanghe (Site Z, 39° 25′ N, 123° 3′ E), respectively (Fig. 1). Triplicate water samples were monthly collected in pre-washed polyethylene bottles at 3 m depth where the scallops were suspension-cultured. Thirty scallops were randomly collected from suspended cages for the measurement of wet weight, and another nine scallops were dissected to detect the glycogen content of adduct muscle.

### Measurement of environmental parameters

Water temperature, salinity and pH were recorded with an YSI meter (YSI Incorporated, USA) at the two sampling sites. The concentrations of nutrients (including ammonium, nitrate, nitrite, phosphate and silicate) and chlorophyll *a* (Chl-*a*) were measured using spectrophotometry [11, 23]. The dissolved inorganic nitrogen (DIN) was defined as the sum of the ammonium, nitrate and nitrite concentrations. The stoichiometric ratios of N/P, Si/P and Si/N represented the molar ratios of DIN to phosphate, silicate to phosphate and silicate to DIN, respectively.

### Phytoplankton DNA extraction and high-throughput sequencing

The genomic DNA extraction and high-throughput sequencing of phytoplankton were performed according to the previous description [47]. The phytoplankton samples were collected by filtering seawater samples with 0.22 μm pore-size membranes (Sagon Biotech, China). The total DNA on membranes was extracted using the Water DNA Kit (Omega, USA) following the manufacturer’s instruction. The quality of DNA extracts was determined by agarose gel electrophoresis, and the DNA concentration was measured using a NanoDrop spectrophotometer (Thermo Fisher Scientific, USA). High-throughput sequencing of 18S rDNA V4 region was performed at Novogene Co., Ltd. (Beijing, China) using the Ion S5 XL platform.

### Analysis of phytoplankton structure

The amplicon sequencing data were analyzed using QIIME 2 (version 2019.7, https://qiime2.org). Single-end demultiplexed reads were denoised into sub-operational taxonomic units (sOTUs) using the deblur plugin in QIIME 2 [2]. Taxonomic classification of sequences was assigned to sOTUs using a Naïve Bayes classifier trained on the SILVA v132 99% OTU database (https://www.arb-silva.de), where reference sequences only included the V4 region of 18S rDNA. Only sOTUs assigned into phytoplankton were retained in the dataset. Resulting taxonomic relative abundances from triplicate samples were grouped together and displayed using stacked bar plots.

Phylogenic tree was constructed by FastTree to analysis phylogenetic diversity [30]. The feature table was rarefied to the lowest number of sequences found in the sample dataset prior to the diversity analysis. α- and β-diversity metrics including Chao1 index, Shannon diversity index and Bray–Curtis distance were calculated using q2-diversity plugin. Principal coordinate analysis (PCoA) based on the Bray–Curtis distance was performed to visualize the dissimilarity in phytoplankton community compositions of different samples.

### Analysis of scallop glycogen content

The glycogen content of adductor muscle was determined using a liver/muscle glycogen assay kit (Nanjing Jiancheng Bioengineering Institute, China) according to the manual. The tissues from three scallops were mixed together as one sample, and there were three parallels for the analysis. In brief, the weighed tissues were suspended in alkaline liquor, and hydrolyzed at 100 °C for 20 min in a water bath. After cooling, the hydrolysate was diluted by adding distilled water and then incubated with color reagent at 100 °C for 5 min. The optical density (OD) value of each sample was measured at 620 nm. The glycogen content was expressed in mg/g wet tissue weight.

### Statistical analysis

Nutrient limitation was determined by both the concentrations and stoichiometric ratios as described by Liang et al. [22]. The minimum concentration threshold for phytoplankton growth was set as 1.0 μM for DIN, 0.1 μM for phosphate and 2.0 μM for silicate. Stoichiometric limitation was identified based on the following criteria: (a) nitrogen limitation, if N/P ratio < 10 and Si/N ratio > 1; (b) phosphate limitation, if N/P ratio > 22 and Si/P ratio > 22; and (c) silicate limitation, if Si/N ratio < 1 and Si/P ratio < 10.

Relationships between phytoplankton community and environmental factors were analyzed by CANOCO 5.0. The environmental factors including water temperature, ammonium, nitrate, nitrite, phosphate and silicate were used as the explanatory variables. The phytoplankton data were first examined by detrended correspondence analysis (DCA) to determine the ordination method [16]. Linear model-based redundancy analysis (RDA) was chosen since the maximum gradient length was shorter than 3.0 [21].

The quantitative data were expressed as mean ± standard deviation, and calculated using one-way analysis of variance followed by a Turkey's multiple comparison. In addition, the two-sample *t*-test was used to compare the data between sites Q and Z. Statistical analysis and graph drawing were conducted using Graphpad Prism 8 and R software (https://www.r-project.org). The level of significance was set at *p* < 0.05.

## Supplementary Information

**Additional file 1: Figure S1.** Temporal variation of salinity (**A**) and pH (**B**) at sites Q and Z.

**Additional file 2: Figure S2.** Succession of Dinophyceae communities at site Q (**A**) and Z (**B**). Phytoplankton composition was represented at level 7 of the taxonomic hierarchy in SILVA v132 release. The top ten abundant groups were shown in the figure and the rest was indicated as “Others”.

## Data Availability

All the sequencing data have been deposited at the NCBI BioProject repository under Accession Number PRJNA803164.

## Abbreviations

Q: Qinhuangdao
Z: Zhuanghe
DIN: Dissolved inorganic nitrogen
N/P ratio: The molar ratio of dissolved inorganic nitrogen to phosphate
Si/P ratio: The molar ratio of silicate to phosphate
Si/N ratio: The molar ratio of silicate to dissolved inorganic nitrogen
Chl-*a*: Chlorophyll *a*
sOTUs: Sub-operational taxonomic units
PCoA: Principal coordinate analysis
RDA: Redundancy analysis
OD: Optical density
DCA: Detrended correspondence analysis
