# Supplementary figures and images for: Dynamics of phytoplankton community in scallop farming waters of the Bohai Sea and North Yellow Sea in China
Source: BMC Ecol Evol. 2022 Apr 15;22:48. doi: 10.1186/s12862-022-02002-z (PMC9013024; doi:10.1186/s12862-022-02002-z)

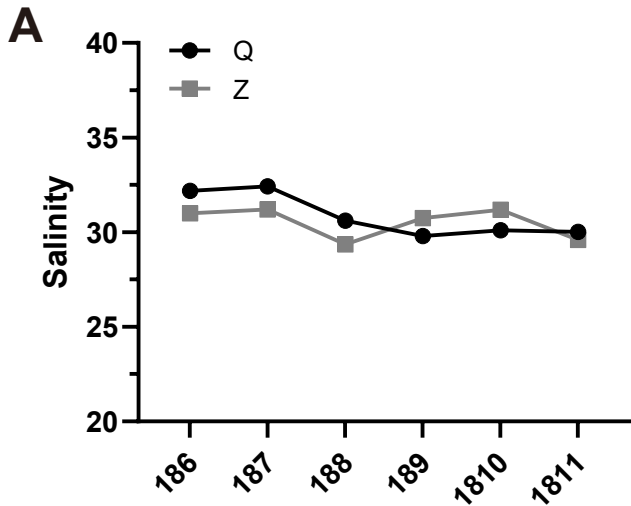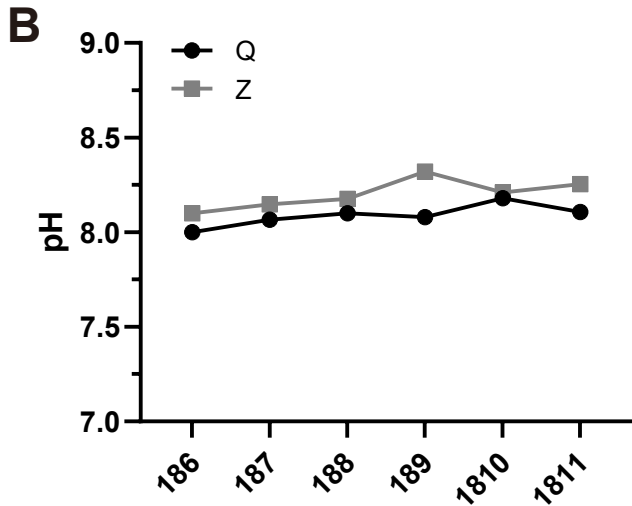

Supplement: Supplementary file 1 — Additional file 1: Figure S1. Temporal variation of salinity (A) and pH (B) at sites Q and Z. [file 12862_2022_2002_MOESM1_ESM.pdf]

**A**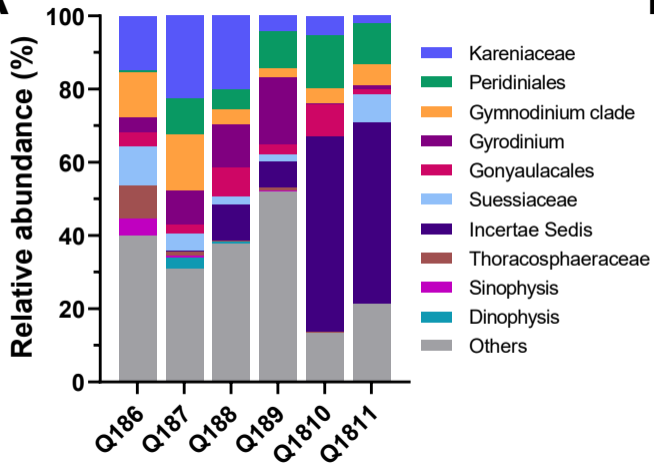**B**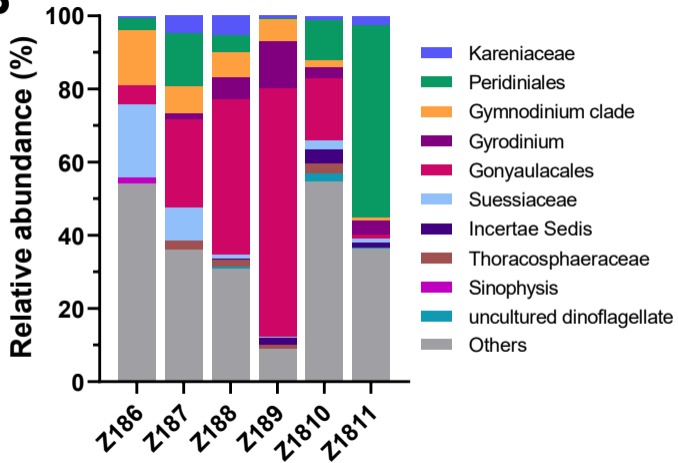

Supplement: Supplementary file 2 — Additional file 2: Figure S2. Succession of Dinophyceae communities at site Q (A) and Z (B). Phytoplankton composition was represented at level 7 of the taxonomic hierarchy in SILVA v132 release. The top ten abundant groups were shown in the figure and the rest was indicated as “Others”. [file 12862_2022_2002_MOESM2_ESM.pdf]
